# Model Organisms and Traditional Chinese Medicine Syndrome Models

**DOI:** 10.1155/2013/761987

**Published:** 2013-12-05

**Authors:** Shuang Ling, Jin-Wen Xu

**Affiliations:** Murad Research Institute for Modernized Chinese Medicine, Shanghai University of Traditional Chinese Medicine, Shanghai 201203, China

## Abstract

Traditional Chinese medicine (TCM) is an ancient medical system with a unique cultural background. Nowadays, more and more Western countries due to its therapeutic efficacy are accepting it. However, safety and clear pharmacological action mechanisms of TCM are still uncertain. Due to the potential application of TCM in healthcare, it is necessary to construct a scientific evaluation system with TCM characteristics and benchmark the difference from the standard of Western medicine. Model organisms have played an important role in the understanding of basic biological processes. It is easier to be studied in certain research aspects and to obtain the information of other species. Despite the controversy over suitable syndrome animal model under TCM theoretical guide, it is unquestionable that many model organisms should be used in the studies of TCM modernization, which will bring modern scientific standards into mysterious ancient Chinese medicine. In this review, we aim to summarize the utilization of model organisms in the construction of TCM syndrome model and highlight the relevance of modern medicine with TCM syndrome animal model. It will serve as the foundation for further research of model organisms and for its application in TCM syndrome model.

## 1. Introduction

It has been found in the early 20th century that the most basic question of life can be answered in the simplest and easiest available biological systems. Simple organisms usually have less cell number and simple distribution of species, which can be observed and manipulated with ease. Since all creatures evolved from a common ancestor, cells have an identity in the basic pattern of development, and the structures and functions of vital genes for life activity are conserved. Therefore, these simple organisms can be used as the models easily to understand the universal law of life world according to their basic biological information. These organisms are commonly referred to as model organisms. As elementary materials for life science, model organisms can not only reveal the fundamental phenomena of life, but also can be an important reference for the exploration of the mechanisms and treatments for human diseases. In recent years, with the completion of genome sequencing, the studies on model organisms have gained a significant development so that human genome research has also entered the “postgenomic era”. The functional genes of model organisms could be directly used for drug discovery and development, agricultural industry and medical diagnosis, and treatments with rapid, efficient, and large-scale identification. Comparative medicine and genetic engineering technology also offered the thought and method for the establishment of human genetic disease models in model organisms, which will be beneficial for the study of unknown functional genes and the development of disease therapy.

Although the application and acceptability of traditional Chinese medicine (TCM) increased in Western countries, the modernization of TCM has remained slow. Due to its worldwide application and potential impact on healthcare, a scientific evaluation system of TCM is necessary. Suitable animal models play an important role in the studies on fundamental theory of TCM and herbal pharmacology. In ancient China, animals were used in drug evaluation a long time ago. Domestic animals (horse, cattle, sheep, pig, dog, or chicken) had been used as bone-fracture models to explore the functions of red copper chippings on bone setting [1]. Broomcorn millet and polished glutinous rice in the absence of vitamins were fed to cats and dogs to observe joint diseases. Rabbits were also used during the study of brain function through acupuncture.

Nowadays, TCM animal model research has become one of the fastest growing areas of modern TCM. It brings positive methodology and modern biological conception and has a positive impact on the development of TCM.

## 2. August Krogh Principle and Model Organisms

Good research system is the key to the success in scientific research. In the field of biomedicine, August Krogh principle [2] interprets that “for such a large number of problems, there will be some animals of choice, or a few such animals, on which it can be most conveniently studied" [3].

The principle requires that scientists should select an appropriate experimental object during studying a scientific problem. Usually, an ideal object in nature is easier to achieve the desired results, or even unexpected discovery when studied on this ideal organism, cell, gene, or protein, thus leading to another concept—model organisms.

Model organisms are special species that have been chosen for certain research aspects. A large amount of information with regards to other species including humans is obtained from model organisms, which will provide valuable data for the analysis of normal human development, gene regulation, genetic diseases, and evolutionary processes. Major model organisms are *Bacteriophage*, *Escherichia coli*, *Saccharomyces cerevisiae*, *Caenorhabditis elegans*, *Drosophila melanogaster*, *Danio rerio*, *Mus musculus,* and *Arabidopsis thaliana*.

## 3. History of Traditional Chinese Medicine Experimental Animal Model [4]

TCM has been practiced for more than 3000 years and has been accepted by a large portion of the population in China as a complementary therapy. *Zheng* (TCM syndrome), the key concept in TCM, refers to the pathological generalization in a certain stage of disease development. Nowadays, TCM is being accepted by more and more Western countries. However, the standards and weak point of fundamental research have restricted its further development. Its safety and pharmacological functions are also uncertain. According to the current status and problems, TCM modernization is highly necessary and urgent.

Since the 17th century, animals have played a vital role in every major medical advance. Experimental studies of TCM animal model started in 1960s in China. Kuang et al. [5] have found that excessive adrenocortical hormone aroused *Yang* asthenia in mice. At the same time, tongue image group of Shanghai Second Medical College has carried out the studies on the pathological change of tongue picture in rabbit models with *Qi-*deficient syndrome (artificial chronic anemia) and *Yin-*deficient syndrome (top digit small intestine side fistula). It is the first time to name TCM symptom. In 1974, abdominal blood coagulation model was used as Blood-Stasis model to explore the pharmacological actions of ectopic pregnancy prescription [6].

After 1976, TCM syndrome model transferring from the disease to *Zheng* was developed. The concept “syndrome differentiation” was focused on the establishment of animal models. In 1977, effects of *Yin*-nourishing drugs and *Yang*-tonifying drugs on *Yin*-deficient syndrome model and *Yang*-deficient syndrome model were explored through histochemistry and molecular biology method. At the same time, Blood-Stasis model and spleen-deficiency syndrome model were also established. These three kinds of models such as Kidney-deficient syndrome, spleen-deficient syndrome and Blood-Stasis syndrome have opened the ancient river of Chinese medicine syndrome [4] model and become the critical research topics for a long time.

In the mid-1980s, a large number of modern technologies such as biochemical immunological techniques, cell culture techniques, DNA analysis, cell fusion techniques, electron microscopy, and image analysis have been used in syndrome model study for achieving objective and standard indexes. Systematic studies of physiological and pathological changes in the syndrome model were conducted *in vitro* and* in vivo* to establish the correlation between modern medicine indexes and Chinese medicine syndrome [7–10].

Through the efforts of more than 40 years, the TCM syndrome model is on the right track and has gradually matured. Nowadays, high throughput genomics, proteomics, and metabolomics provide a new technological platform for the substantial fundamentals of diseases, syndromes, and prescriptions [11, 12].

In recent years, people realized that the unique theory of holistic concept and syndrome differentiation of TCM is coincided with the view of system biology [13]. Due to the combination of theory and technology in modern system biology, the gene expression, protein expression, and metabolic profiles of syndrome can be easily achieved and the relationship between profiles and syndrome can be understood in the aid of bioinformatics techniques, which will provide a proper way to reveal the nature of TCM syndrome.

## 4. TCM Syndrome Model and Disease Model

*Zheng* is a key concept peculiar to disease recognition in TCM. It is an indicator of the integrated reactive state of disease essence; it summarizes the cause, location, nature, and trend of the diseases as well as body's defense in the dynamic evolution process.

The “Disease” and “*Zheng*” recognize the disease essence from different angles [14], but there are also some important differences. A mature disease model always has some accurate special pathological features for distinguishing other diseases. For example, asthma is the common symptom of chronic asthmatic bronchitis and bronchial asthma, but the differences among the age of onset and the history of cough and sputum could distinguish them through the analysis of symptoms.

The characteristic of *Zheng* such as fuzzy, dynamic, complex, and nonspecific determines the complexity and diversity of TCM syndrome models. A disease process can be divided into various types according to syndrome differentiation; and the same TCM syndrome model occurs in many disease processes. For example, angina, called *XIONG BI* (chest impediment and heart pain) can be divided into 7 TCM syndromes such as *Qi* stagnation syndrome, Blood-Stasis syndrome, phlegm-turbid syndrome, congealing cold syndrome and deficiency of heart *Qi*, *Yin,* and *Yang *syndromes. A typical TCM syndrome, Blood-Stasis syndrome, described in TCM theory as a slowing or pooling of the blood, can be observed in many diseases, such as coronary diseases, heart failure, stroke, hyperlipemia, diabetes, rheumatism, endometriosis, and even depression. But above all, disease model and TCM syndrome model are closely related. On the precondition of disease differentiation, syndrome differentiation is more significant in disease diagnosis and treatment and TCM symptomatology study.

TCM syndrome model is the basic tool for the studies on both dynamic development of TCM symptoms and pharmacology of Chinese herbal formula. However, the replication of animal models that can reflect characteristics of *Zheng* is as a premise, which accurately obtains laboratory findings of traditional Chinese medicine. There are five kinds of TCM syndrome animal models: pathogenic models, pathological models, drug-induced models, models of TCM etiology combined with pathology, and models of diseases combined with TCM syndrome. In a variety of ideas developed in TCM syndrome animal models, the models combined with disease and TCM syndrome have both pathological features of Western diseases and characteristics of *Zheng*. On the biological basis of the unclarified *Zheng*, the models combined with disease and TCM syndrome are more appropriate models in the case study.

A major challenge for exploring the mechanisms of human diseases is the selection of an appropriate animal model that accurately reflects the disease. Mechanistic studies in animal models may provide important information on the processes of human disease. Therefore, animal model (Western medical model and TCM syndrome model) is a useful tool for drug development and fundamental research. Nevertheless, TCM syndrome model has its unique applications because of the different philosophical thoughts and medical systems of Western medicine and traditional Chinese medicine. TCM syndrome model is the basic tool for the studies on both dynamic development of TCM symptoms and pharmacology of Chinese herbal formula. A good TCM syndrome model is described, which can bridge the relationship between the essence of relevant TCM syndrome in the clinic and the experimental data to diagnostic criteria of TCM syndrome animal model. Nowadays, the establishment of TCM syndrome model evaluation system is still in the beginning phase. But in the impulse of development of TCM and the government's recognition, there has been made a rapid progress in this field. To date, TCM syndrome animal models have a variety of different evaluation methods, such as macroeconomic performance of the model animal, individual physical and chemical indicators, TCM syndrome differentiation through formula effect assessment, according to modeling factors presumably identified, equivalently corresponding to clinical diagnostic criteria. In view of the close relationship between disease and *Zheng*, some scholars first proposed the 3D establishment and assessment of the combination of disease and TCM syndrome animal model [15]. Based on the clinical syndrome diagnostic criteria, macroscopic indexes of animal model, microscopic indexes of genomics, proteomics and metabonomics, and at last, syndrome differentiation through formula effect assessment will be implemented to confirm this program. The first information gathering workstation of four diagnostic methods is established based on long observation of the animal [16]. It sets up the framework of animal four diagnostic methods and TCM syndrome differentiation. Objectification, standardization, and quantification have been successfully introduced into the realm of TCM symptomatology study.

## 5. Establishment of TCM Syndrome Model

Most essential thoughts of TCM theory are dialectical way, which is stemmed from the ancient Chinese philosophical thoughts, for example, the theories of *Yin-Yang*, Five Elements, *Zang-Fu* (viscera), Channels-collaterals (meridians), *Qi*, and Blood and body fluids. *Zheng* or TCM syndrome is the basic unit in traditional Chinese Medicine. The whole therapeutic system is based on the classification of TCM syndromes, which is a critical strategy to understand and diagnose diseases by TCM. Similarly, under the guidance of a variety of TCM theories and principles, this dialectical approach has been used in animal models with TCM syndromes. TCM syndrome models were usually used in the study of relevant syndrome essence or the function of herbal recipe.

**Figure 1:**
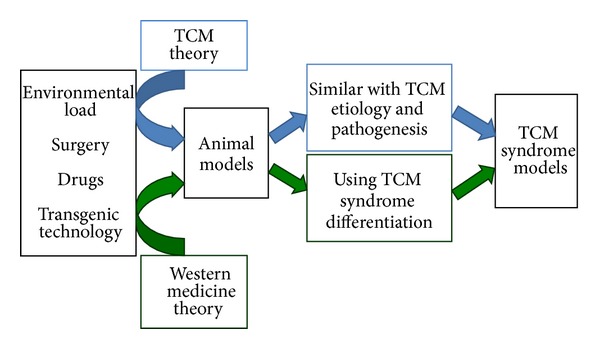
Approaches and applications of current TCM syndrome models.

Figure 1 summarizes the current approaches and applications of TCM syndrome models. Part 1, under the guidance of TCM theory, attempts to apply the environmental loading, surgery, hormone, or drug delivery to establish the syndrome animal model. Part 2, on the basis of currently available animal models of diseases in the field of Western medicine with the combination of TCM theory, lists the identified approaches to establish animal models. Due to the similarity between clinical symptoms and TCM syndromes, these models are usually used directly as a model of syndrome.

TCM syndrome models have common points with modern models. Some TCM syndrome models are exactly same as modern models including stomach ache, cancer, or diabetes. Other TCM syndrome models share similar pathogenic factors or pathological changes with modern models. Currently, the models of disease combined with TCM syndrome have become the mainstream method in the studies of TCM syndrome models.  Table 1 summarizes the types, characteristics and application of TCM syndrome models. The animal model of modern disease is in conformity with the clinical practice, in which the evolutionary process of TCM syndrome can be observed.

Furthermore, by studying modern models and TCM syndrome models, researchers can learn a lot regarding human disease and health problems. From a clinical viewpoint, animal models represent the nature of diseases despite the different philosophical thoughts and medical systems. We can take advantage of animal models for basic science study, clinical research, and drug development.

### 5.1. TCM Syndrome Model Based on TCM Etiology and Pathogenesis [33]

On the basis of TCM theory, the pure TCM syndrome model is not equal with the modern medical model, such as *Yang*-deficient syndrome, Kidney-deficient syndrome, Blood-Stasis syndrome and *Cold* syndrome.

#### 5.1.1. TCM Syndrome Models Based on TCM Diagnostic Methods

*Zheng* is identified from four main diagnostic TCM methods: observation, listening, questioning, and pulse. Tongue picture with abundant information can be observed easily. Consequently, it is very important to realize the tongue characterization in animal models. For example, red tongue model indicating the *Heat-*syndrome usually can be induced by long-term treatments with *Heat* drug (rats were treated with water extract of ginger, *Radix aconiti, Lateralis preparata, *and* Cinnamomum cassia* for 26 weeks) [17]. Thin white greasy tongue fur implying the impairment of the stomach can be copied with alcohol (50% v/v), overeating, and eating disorders [18].

#### 5.1.2. TCM Syndrome Model Based on Syndrome Identification of Eight-Principle

The eight-principle is a general guideline of all kinds of syndrome type methodologies, which generalize the balance of *Yin-Yang*, interior or exterior environments, equilibrium between *Cold* and *Heat*, and deficiency or excess in the *Zheng*. Eight-principle serves the guides for the identification of all syndromes, such as the location or severity of pathological changes, the nature of illness, the condition of body resistance, and pathogenic factors. Different components have specific metabolic features and different susceptibility to certain diseases. For example, *Wind*-*cold* environment is one of the reasons for *cold* in the exterior syndrome. Excessive exercise after oral administration of *Heat* drugs, such as ginger, *Radix Aconiti, Lateralis preparata, *or* Cinnamomum cassia*, causes *Heat* syndrome due to insufficiency of *Yin*-fluids in animal models (rats were treated with water abstract of *Heat* drugs for 28 days and swimming for 5 min before sacrifice) [19].

#### 5.1.3. TCM Syndrome Model Based on *Qi*-Blood-Liquid-Fluid Pattern Identification

*Qi*, Blood and Body Fluid are a substantial basis for the functional activities of life in TCM theory. *Qi*-deficiency mice model can be prepared by starvation (controlling forage amount in 125 g/kg^−1^·d^−1^ for 14 consecutive days) [20]. ^60^CO *γ*-ray radiation (3.5 Gy in 4 miles) or cyclophosphamide (120 mg/kg i.p.) can result in the damage of the blood-generating function of bone marrow, which is the representative of blood-deficiency syndrome [21].

#### 5.1.4. TCM Syndrome Models Based on Viscera Syndrome Types

The Viscera theory, also named as *Zang-Fu* theory, was formed in the “Shanghan Lun”, a famous and authoritative book of Treatise on Febrile and Miscellaneous Diseases in the Han Dynasty. The main point of this book can be traced back in the “Huangdi Neijing” (Inner Canon of Yellow Emperor) in the Spring and Autumn Period of China (400 B. C.). *Zang* and *Fu* are composed of the organs of five *Zang* and six *Fu*. Five *Zang* includes the organs such as heart (including the pericardium), lung, spleen, liver and kidney. Six *Fu* include gall bladder, stomach, large intestine, small intestine, urinary bladder, and Sanjiao (triple energizer). According to the TCM theory, *Zang* and *Fu* are not the simple anatomical concepts and are not equal to the anatomical organs from Western medicine. However, they are the important representation of physiological functions and pathological changes in human body. TCM treatment is characteristic of the analysis of the entire system and has the focuses on the balance of *Yin-Yang* through readjusting the functions of *Zang-Fu *organs. Therefore, a suitable animal model for the classification of viscera syndromes plays an important role in the understanding of visceral connotations, disease prognosis, and corresponding diagnosis and treatment methods.

At present, there are many commonly used models in TCM experimental research. For example, pulmonary *Qi*-deficiency syndrome refers to asthenia syndrome due to the insufficiency of *Qi* and hypofunction of the lung. These symptoms are usually observed in chronic obstructive pulmonary diseases.

Heart-Blood-Stasis syndrome is a more common syndrome differentiation of heart disease. Experimental animals can be established myocardial infarction model by ligation of the anterior descending branch of the left coronary artery or continuous injection with isoprenaline (100 mg/kg) [22, 23].

The clinical symptoms of spleen and stomach diseases in TCM are usually poor appetite, abdominal distension, loose stool, nutrition deficiency, heaviness sensation of arms and legs, lassitude, and emaciation. Low-protein diet with lapactic herbs or subcutaneous injection of reserpine can simulate vague, systemic, chronic, and deficient clinico-pathological features of spleen-deficient syndrome [24].

The kidney is an extremely essential organ in the whole process of life. The most important function of the kidney is the essence storage of life in TCM theory, which is the foundation of the capability for reproduction, growth, and development. Kidney diseases are mainly involved in the deficiency of kidney. Aging, hereditary insufficient, intemperance of sexual life, and disorders of viscera, especially spleen and other reasons lead to asthenia or deficiency of *Qi*, *Yin*, *Yang,* or essence in kidney. Syndromes of Kidney-*Yang* deficiency are usually observed in hypothyroidism, hypoadrenocorticism, hypogonadism, and chronic nephritis. Therefore, excessive administration of hydrocortisone acetate, bendazole, hydroxycarbamide, thyroidectomy, or adrenalectomize can induce Kidney-*Yang* deficiency syndrome in model animals [5]. Syndromes of Kidney-*Yin* deficiency are commonly observed in some chronic consumptive diseases, such as advanced cancer, chronic nephritis, liver cirrhosis, diabetes, and tuberculosis. The administration of adrenocortical hormone and thyroid hormone in large doses or medicinal herbs with heat potency in TCM property, such as aconite, curculigo, orchioides, cinnamon, or epimedium, can induce Kidney-*Yin* deficiency syndrome in the animal model.

Liver disease mainly manifests in abnormal changes in storing- and dispersing- blood and the disorder of Liver-*Qi*. For instance, Liver-*Qi* stagnation may bring irregular menstruation and mental depression. Hyperactivity of Liver-*Qi* may increase your risk of suffering from irritability and anger. The digestive function of spleen-stomach and the excretion of bile are also affected by the syndromes of jaundice and bitter taste. Dizziness, tremor of limbs, numbness of hands, shaking head, and even sudden coma or hemiplegia is associated with the abnormal function of the liver. Liver-*Qi* depression model can be established by bandaging or irritating animals [34–36]. In TCM diagnosis, the 2K2C (two-kidney two-clip) renovascular hypertensive model is similar to the process of Liver-*Yang* Forming *Wind*-Syndrome (LYFWS), which refers to Wind syndrome due to the hyperactivity of Liver-*Yang*, usually observed in hypertension, cerebral hemorrhage, cerebrovascular accident sequela, Parkinson's disease, epilepsy, and injury of spinal cord [37]. Chronic hepatic injury induced by carbon tetrachloride (hypodermic injection of 40% oil solution, 0.3 mL/100 g body weight for 6 weeks) plus heat herbal compound prescription (rats were treated with water extract of ginger, *Radix aconiti, Lateralis preparata, *and* Cinnamomum cassia* for 2 weeks) can decrease body weight and increase heart rate and temperature of experimental rats [38], which is consistent with clinical signs of Liver-*Yin* deficiency syndrome.

### 5.2. TCM Syndrome Models Based on the Etiology and Pathology of Western Medicine

According to TCM theory, a part of diseases such as cancer, stroke, myocardial infarction, and diabetes share similar etiology and pathogenesis identified by modern medicine. Thus, the models usually used by Western medicine seem to be directly applicable.

For instance, liver cancer can be induced by chemical carcinogens such as diethylnitrosamine (DEN), dimethylamino-azobenzene (DBA), *o*-aminozaotoluene (OAAT), 2-acetamidobenzoic acid (2AAT), and aflatoxin in rats. Stomach ache, a common syndrome of stomach disease, is usually observed in acute gastric ulcer induced by oral administration of 1% formalin or salicylic acid [25]. The usual clinical symptoms of lung disease include cough, dyspnea, and lung distension. Pneumonia, emphysema, and pulmonary fibrosis can be induced by infusing bacteria, papain, and bleomycin through tracheal intubation, respectively. Streptozotocin (STZ) plus high calorie and high sugar diet cause diabetes, so called emaciation-thirst disease during TCM diagnosis. Whether strokes by middle cerebral artery occlusion or thoracic obstruction by the left anterior descending coronary artery ligation, both present Blood-Stasis syndrome. For example, chronic myocardial ischemia model generated by ameroid constriction of a coronary artery shows typical Blood-Stasis syndrome with clinical signs of dark purple tongue, arrhythmia, coronary stenosis or obstruction, and increased blood viscosity [26]. This kind of model is highly reproducible and easy to establish. It is an ideal model for the study of TCM theory through the thought of Western medicine.

### 5.3. TCM Syndrome Models Integrated with Traditional Chinese and Western Medicine

Another ideal model called combination of disease and syndrome mode (CDSM) is usually used to evaluate the preclinical validity of TCM. *Zheng* is induced according to the pathogenesis of TCM theory on the basis of Western medicine disease models.

For example, type 2 diabetic animals supplemented with 0.1% prednisolone (0.1 mL for 13 days) and 0.1% adrenaline injection (0.1 mol for the last day) for increasing blood lipid and intimal thickening, and causing early plaque formation, higher hematocrit, and cardiovascular morphological changes, can reveal a success Heart-Blood-Stasis syndrome model [27]. Moreover, subcutaneous injection with large dose of 0.1% adrenaline (0.08 mL/100 g body weight) causes peripheral circulatory disturbance, and then animals are placed in an ice-water bath to simulate the syndrome of Congealing-*Cold* with Blood-Stasis [28]. On the other hand, in order to build an animal model of Heart-*Qi* deficiency syndrome, a commonly used method is dietary restriction combined with forced load swimming and propranolol (0.5 mL, 1 mg/mL) or pituitrin injection (0.2 mL, 5 U/mL), resulting in the myocardial damage [39]. To sum up, CDSM promotes the understanding of diseases, *Zheng,* and relationship between *Zheng* and diseases, which is beneficial to the application in clinical discipline of Chinese and Western integrative medicine.

### 5.4. TCM Syndrome Models from Genetically Engineered Animals

With the development of functional genomics and model organisms, genetically engineered animal model is one of the most powerful research systems in the area of life science. Genetic engineering technology, including gene targeting, gene silencing, and transgenic technique, has been used to induce a variety of genetically engineered animals.

Mouse is the closest mammalian model organism to humans. The application of transgenic technology to modify the mouse genes has become commonplace. Transgenic animals also offer the opportunity for pharmaceutical research of TCM. Mutations of *Adenomatous polyposis coli* (*Apc*) gene are important in sporadic colorectal tumorigenesis. The first mutant in *Apc *gene in mice came from a colony of randomly mutagenized mice. The mutant model of mouse tumor suppressor genes can cause similar symptoms with human cancer. The *Apc* mutant model mouse provides an *in vivo* environment to evaluate the validity of drugs.

From clinical symptoms, based on TCM theory, gene knockout mice can be considered as the model for naturally inherited insufficiency and hypoplasia. For example, *ApoE* gene knockout mice were used as Phlegm-Stasis syndrome model in atherosis and dementia [29]. Knockout mice of *Nurrl *gene, a transcription factor, are associated with Parkinson's disease that is a common degenerative disorder of the central nervous system in the elderly. The *Nurrl *knockout mouse model can be used in the studies on *Chan-Zheng*, a clinical syndrome of TCM characterized by tremors, muscle rigidity, and flaccid [30].* db/db* mutant mouse is a kind of diabetic model, because Lepr^*db*^ is an autosomal-recessive mutation on chromosome 4 and displays the characteristics of obesity, hyperglycemia, high insulin secretion, polyphagia, and polyuria, which are similar to non-insulin-dependent diabetes in humans. The *db/db* mutant mouse also provides an ideal model of diabetic microvascular disease, which presents the characteristics of a typical *Yin*-deficiency and Blood-Stasis syndrome. The tongue color of C57BL/6J-HBV transgenic mice, a chronic hepatitis animal model, is mostly purple due to microcirculation disturbance. The change of tongue color implies the severity of illness and the degree of Blood-Stasis syndrome [31]. The APP transgenic mice exhibit many pathological changes of Alzheimer's disease include extracellular A*β* deposition, synaptic and cognitive defects, reactive astrocyte hyperplasia and dystrophic neurons, which are also commonly used in the study on senile dementia in TCM [40]. HLA-DR4 is associated with rheumatoid arthritis and multiple sclerosis. The Abb knockout/transgenic HLA-DR4 mice are susceptible to arthritis and connective tissue diseases, which is similar to arthromyodynia (or *Bi* syndrome), a pathological phenomenon of Cool-Dampness of joints according to TCM theory and used to analyze the correlation between TCM syndromes and rheumatoid arthritis [32].

## 6. Diagnostic Criteria for TCM Syndrome Animal Models and Biomarkers

There is a difference between animal models of TCM syndromes and Western medicine, which is established on the basis of clear pathological changes; on the contrary, dynamic *Zheng* is the soul of TCM syndrome animal models. *Zheng* is the external manifestation of diseases in some stages with a dynamic evolution; however, the mechanisms are still not clear. Because Chinese medicine is personalized medicine, the majority of the diagnostic criteria are still in the initial stage of the establishment and evaluation of animal models according to TCM syndromes and also lack widely accepted “gold standard”; therefore, the replication and effective evaluation of these animal models are still limited. During the evaluation of the syndrome model, several aspects of these works are still to be improved.

### 6.1. Establishment of TCM Syndrome Diagnostic Criteria

Macrosyndrome differentiation is a traditional method of Chinese medicine diagnosis through macroscopic information from observation, auscultation, smelling, asking, and pulse. This method is influenced by subjective factors, which can be applied to the evaluation of TCM syndrome animal models. Currently, a growing emphasis on microsyndrome differentiation considered by Western idea to identify diseases through detectable or quantifiable disease-related laboratory indicators as a basis for judging TCM syndromes or diagnostic criteria is present. The development of genomics, proteomics, and metabolomics technology plays a catalytic role for the exploration of TCM essence. The application of these methods can provide “TCM syndromes-related biomarkers” to improve the diagnostic criteria of syndrome animal models.

For example, the study [41] on patients with diabetes indicated that glucose, inositol D, C4 sugar 2, and C4 sugar 1 may be the new biomarkers for diabetes. However, the changes in level of xylose and C4 sugar 2 present the opposite results in diabetic patients with deficiency- or excess-syndrome, which they can serve as biomarkers to distinguish between deficiency- or excess-syndrome. Furthermore, compared with *cold* syndrome and *heat* syndrome in gastritis, Rui Li and his colleagues [42] found that leptin is a biomarker of *cold* syndrome, suggesting a low energy metabolism. On the contrary, CCL2/MCP1 is a biomarker of *heat* syndrome, showing increased inflammation, body temperature, metabolism, and immune regulation. These biomarkers will help for distinguishing *cold *from *heat* syndromes in gastritis model. Some studies have also reported [43, 44] that different types of Phlegm-Stasis syndrome induced by hyperlipidemia and atherosclerosis, dividing into 3 subtypes of phlegm-syndrome, Blood-Stasis-syndrome, and phlegm-and-Blood-Stasis-syndrome, have specific changes in plasma proteins; for example, (1) the levels of fibrinogen B-chain and apolipoprotein AI precursors can be used for distinguishing Phlegm-Stasis syndrome; (2) fibrinogen C-chain, albumin, and apolipoprotein AI precursors can be used as biomarkers to distinguish phlegm-syndrome and phlegm-and-Blood-Stasis-syndrome; (3) the application of indicators such as haptoglobin precursor, adrenomedullin-binding protein precursor, albumin, and complement C4 are able to distinguish phlegm-and-Blood-Stasis-syndromes. The variation of these biomarkers can result in the changes in protein expression, thus leading to the changes in the Phlegm-Stasis syndrome among different types.

### 6.2. Established Evaluation Methods for the Syndrome Characteristics of Animal Models

At present, a large number of scholars have studied and summarized the characterisyics of the TCM syndrome animal model; for example, some people [45, 46] think that there are differences in rat/mouse models in physique and characterization of TCM syndromes. To this end, through noninvasive information collection and analysis on the characterization of small animals, the implementation of individualized diagnosis and treatment of small animals has initially achieved the standardization, objectivity, and quantification of diagnostic methods. The application of intelligent diagnostic techniques, such as, digital cameras, photoelectric blood stream plethysm, infrared imaging, colorimeter detection, and computer image processing, can observe the changes in animal hair luster, body weight, body temperature, heart rate, claw color, tongue color, excrement and urine, secretion and crissum color, and behavior changes including burnout, curled up, keeping warm together, decreased activity, trembling, drowsiness, and carpenter, which is used to judge the types of syndromes of model animals. Some studies [47] have established the scoring criteria and quantification table, a diagnostic scale for the model of liver*-*depression-and-spleen-deficiency caused by chronic restraint stress including a total of 26 indexes such as the appearance of characterization, stress response, feces situation, and general indicators (body weight, eating, drinking, body temperature, and so on).

### 6.3. Syndrome Differentiation through Formula Effect Assessment

The application of pharmacological effects was a widely accepted formula that disproves symptoms lesions and locations of the established animal models, which is also a way to improve the diagnosis. For example, on the above model [48], caused by chronic restraint stress to result in liver-depression-and-spleen-deficiency syndrome, after administration of “Xiaoyao San", a formula with the function of dispersing stagnated liver *Qi* and relieving *Qi* stagnation, nourishing spleen to harmonize with nutrient *Qi* can significantly improve animals' symptoms and result in the reduction of various metabolites from some biomarkers such as lactic acid, choline, N-acetyl-glycoprotein, saturated fatty acids, blood sugar, as well as the enhancement of unsaturated fatty acids and high density lipoprotein, which reveal clinically similar therapeutic effects, suggesting that it is generally successful to establish animal model of TCM symptoms. Moreover, Professor Shen [49] has systematically investigated the essence of kidney-*Yang*-deficiency and believes that the regulation center of kidney-*Yang *deficiency is the hormone axis from the hypothalamus to the top. To this end, he used “Yougui Yin", a classic formula for nourishing kidney-*Yang*-deficiency, to confirm his hypothesis. Experimental results show that “Yougui Yin” can specifically improve the mRNA expression of hypothalamic corticotropin-releasing factor and enhance the role of neuronal excitability.

In TCM animal models, despite numerous efforts, however, the ambiguity and complexity of TCM syndrome differentiation formed different standards of animal models, thus leading to the lack of comparability. This was largely hindered the development of Chinese medicine research; therefore, the establishment of a unified, objective diagnostic criteria is highly urgent.

Currently, the disease and syndrome integrated with animal model have become the mainstream model of the TCM syndrome. First, according to Western diagnostic criteria established disease model, and according to TCM clinical diagnostic criteria to collect animal information characterized by TCM syndromes; then, further application of genomics, proteomics, and metabolomics technology can exert the exploration of microlaboratory diagnostic criteria (such as biomarkers) at the molecular level. After obtaining the objective and reliable data, the application of data mining technology can be used to deal with a huge number and complicated data analysis and to understand their intrinsic linkages and rules; finally, the approval of classic formula can form an evaluation criteria of the diseases and syndrome integrated with animal models.

## 7. Application of TCM Models on Drug Screening and Mechanism Research

Unlike Western medical model, TCM symptom model is based on the guidance of TCM theory with certain characteristics of *Zheng* and human diseases. For this reason, some small mammals such as rats and mice are the most appropriate model organisms [7]. Guinea pig, rabbit, cat, dog, monkey, or mini-pig are also popularly used. Compared with mammals, lower model organisms such as yeast, worms, drosophila, and zebra fish are not suitable because of lacking the carrier for diagnosis and TCM syndrome differentiation. In contrast, due to their similarity with human genes and sharing common biochemical mechanisms or certain disease characteristics, the simple model organisms are still suitable for the use of high throughput screening for bioactive components in Chinese herbals and the exploration of disease mechanisms.

### 7.1. Model Organisms in Drug Discovery

#### 7.1.1. Antiaging Drug

From lower model organisms to higher primates and even human itself, longevity is limited by the interaction of genetic and environmental factors. Regulatory pathways and physiology are relatively conserved because the genetic mechanism of life span is dependent on species. The studies regarding longevity mechanisms have stepped into functional genome stage along with the advancement of technology and development of genome theory. According to long-term clinical practices in TCM, a unique theory of longevity has already achieved; for example, Kidney deficiency is considered as the basic cause of aging. Many antiaging drugs have recorded in ancient herbs, and most of which are Kidney-reinforcing formula. Therefore, the nourishing strategies of kidney combined with other viscera such as invigorating spleen and replenishing *Qi*, or promoting circulation and removing Blood-Stasis according to different syndromes is an ideal antiaging strategy.

Animal model for antiaging drug screening is a promising approach for drug discovery. The characters of* Caenorhabditis elegans *and *Drosophila melanogaster* make them useful for antiaging research because of short generation time, big progeny size, highly detailed genetic maps, and cheap breeding in the laboratory. “Erzhi Pill” and Chuanxiong Extract selected from drug screening on *C. elegans* could prolong the lifespan of the animals from more than 30 kinds of TCM [50]. “Erzhi Pill" is a liver-and-kidney-nourishing formula and Chuanxiong Extract reveals the obvious *Qi*-replenishing and *Blood*-activating effects. Although both formulas have the regulatory functions for insulin/IGF-1 signaling pathway, “Erzhi Pill" can be involved in neuroendocrine genes and clock genes, while Chuanxiong Extract reveals an obvious effect on energy metabolism. In addition to delaying the aging process, Kidney-*Yang*-tonifying herb, *Epimedium*, can also promote the reproductive peak of *C. elegans *[51], as well as kidney-*Yin*-nourishing herb, *Cordyceps militaris*, on* Drosophila melarogaster *[52].

#### 7.1.2. Cardiovascular Drugs

Zebra fish is a popular model for high throughout drug screening. In recent years, the transgenic zebra fish [Tg (flil: EGFP)] has become an important model of angiogenesis research, which is characterized by the expression of green fluorescent protein in endothelial cells of vascular system. The significant effects of the drug on blood vessels can be directly observed. Generally, herbal extract or corresponding components with *Qi*-tonifying and blood-promoting functions can promote angiogenesis in zebra fish model. The antiangiogenesis drugs commonly strengthen healthy *Qi* to eliminate pathogens, which are tested and validated in mammalian models even in clinics. For example, *Qi*-tonifying drug such as *Astragalus* polysaccharide can repair the injured vessel by increasing KDR-l, KDR, and Flt-1 mRNA expression [53], and curcumol can activate blood stasis by VEGFA and VEGFR2 [54]. Water-soluble components of *Angelica sinensis* can also be used in chronic diabetic foot ulcer and angiogenesis [55]. On the contrary, *Angelica sinensis* oil may suppress angiogenesis, induce apoptosis, and activate p38 and ERK1/2 signaling pathway [56]. *Qi*-activating drug such as tangerine peel and blood-cooling drug such as indirubin can inhibit angiogenesis by inducing HUVEC apoptosis and G0/G1 arrest [57].

### 7.2. Mechanisms of TCM Treatments on Neurodegenerative Diseases

Neurodegenerative diseases including Alzheimer's disease, Parkinson's disease, Huntington's disease, and amyotrophic lateral sclerosis refer to the progressive loss of structure or function of neurons in the central nervous system. The current treatment of neurodegenerative diseases is limited. Establishing a neurodegenerative disease animal model for drug screening and mechanism study is of significance.

#### 7.2.1. Parkinson's Disease

Parkinson's disease (PD) is an age-related movement disorder. There are two most prevalent PD pathological markers: the formation of proteinaceous inclusions in PD patient brains and selective loss of dopamine (DA) in neurons. Nonmammalian organisms have been developed to explore cellular mechanisms and the discovery of new drugs. For example, zebra fish larvae are sequentially exposed to neurotoxin, 1-methyl-4-phenyl-1,2,3,6-tetrahydropyridine (MPTP), and Chinese herbal extract. The extract of *Fructus alpinia oxyphylla* and *Flos eriocauli* can prevent and reverse the degeneration of dopamine neurons and improve the deficiency of behaviors. The expression of human *α*-synuclein gene in *Drosophila* may be the model of Parkinson's disease with proteinaceous inclusions and locomotor dysfunction. The organic pesticide rotenone has been found to cause DA neurotoxicity. Both the polysaccharides from *Cordyceps militaris *and the extract of *Acanthopanax senticosus* have shown the protective effect against rotenone-induced oxidative damage in *Drosophila melanogaster* [58, 59].

#### 7.2.2. Alzheimer's Disease

Alzheimer's disease (AD) is a chronic neurodegenerative disorder characterized by cognition impairment and progressive decline in memory, neuronal loss, formation of neurofibrillary tangles (NFTs), and senile plaques. The major relevant gene includes A**β** precursor protein (APP), Presenilin1 (PS1), and Presenilin2 (PS2) genes, tau protein and ApoE gene. In order to study AD pathogenesis *in vivo*, the model systems such as* C. elegans* and *Drosophila* will facilitate to high throughout genetic screening. In addition, the gradual loss of brain cells and A**β** secretion to the extracellular compartment in *Drosophila* with AD were observed. Its pathological features are similar to AD in human. Therefore, the metabolic process of APP and the toxicity of A**β** as well as retinal neuron disease and amyloid deposition can be observed. Turmeric, an ancient Chinese herb used in promoting blood circulation and removing *Ji*-syndromes (a syndrome of *Qi*-Stagnation and Blood-Stasis), has been proved to inhibit the formation of A**β** oligomers and the transformation of A**β** oligomer into fibrils [60].

### 7.3. Safety Evaluation of TCM

Zebra fish has been widely used in embryo, environmental, pathological, and drug toxicological studies. When drugs are added to the living water of zebra fish, and the death rate can be evaluated after 24 h administration, which can be used for the rapid screening and toxicity evaluation of traditional Chinese herbs on zebra fish [61]. A metabolite of mothballs, 1,4-naphthoquinone, can inhibit the activity of apoptotic protein CED-3, thus correspondingly inhibiting cell apoptosis. These findings suggest that mothballs as a daily necessity have carcinogenic tendency [62].

## 8. Conclusion and Prospect

Model organisms play an irreplaceable important role in fundamental studies of modern life science. In the last decades, important scientific discoveries achieved through model organisms are constantly emerging. Now the life science has entered the era of functional genomics. Functional genes of model organisms can be obtained and identified in a large-scale, fast, and efficient way, and can be used directly for drug discovery, disease diagnosis, and disease treatment. Model organism obtained through gene engineering technology and modern comparative medicine has the advantages for exploring the functions of new genes, verifying cellular metabolism or signaling pathways, and improving the development of disease diagnosis and management. Compared with the rapid development of Western medicine, the technology of traditional Chinese medicine lag behind, which has restricted the development of traditional Chinese medicine.

**Figure 2:**
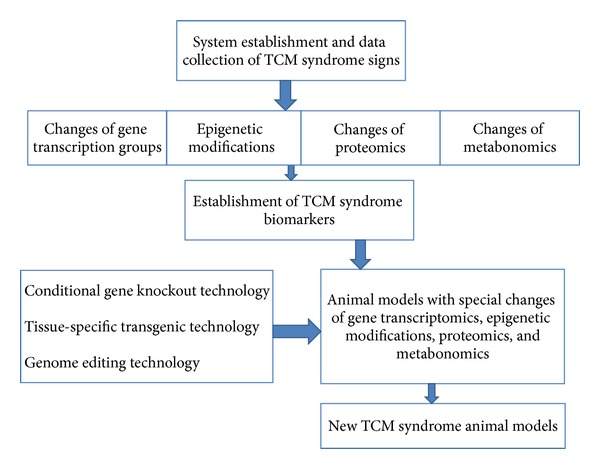
Assumptions regarding the establishment of new animal models for TCM syndromes.

Different constitution types have specific metabolic features and susceptibility to certain diseases. Some studies have shown that a part of TCM syndrome has the genetic [63, 64], proteomic [65, 66], and metabolomic [67, 68] basis according to such constitution classification, which is closely correlated with syndrome types. We need to make full use of achievements in modern science and technology to establish *Zheng* model according to TCM theory, which will be helpful to reveal the essence of TCM syndrome and to promote theoretical creativity. The application of technologies for gain-function or loss-function can benefit for establishing a stable and heritable animal model in accordance with the demand of TCM syndrome. Nowadays, conditional gene knockin, knockout, or transgenic technologies are already mature in the production of model animals. These technologies can be used to determine the temporal and spatial expression pattern on a specific period and in a specific tissue and organ [69–71]. Although previous knockout of rats has difficulty in achieving successful results, recent studies have shown that TALEN approach is a widely applicable technology for targeted-genome editing that can result in the conditional gene knockout in rats [72–74]. This technology is an efficient and rapid method to gain the prospect of conditional knockout rats. Therefore, these technologies can be used to explore specific function or role of genes in specific tissues and organs under certain conditions. Likewise, these technologies also can provide a good prospect for manufacturing of TCM syndrome model that has the requirement of altered gene expression at a given time and specific organs or tissues. The studies on TCM syndromes require animal models with specific genotypes to achieve good reproducibility, uniformity, and stability. These assumptions are shown in Figure 2. These technologies are expected to become an effective way for TCM modernization.

However, due to the difference between animals and humans, the TCM syndrome animal model is complex, and some important symptoms and signs are difficult to reproduce in an animal model, such as tongue pictures, pulse conditions, and emotional symptoms. Transgenic technology is still facing some problems, such as low productivity, long production cycle, inefficient integration of genes, and high cost. Meanwhile, transgenic animals have high mortality and fertility rate. Moreover, there are technical issues also involved in ethical, legal, security, and other issues. How to improve the performance of model organisms and integrate with TCM research still needs further consideration and exploration.

## Figures and Tables

**Table 1:** Summary of TCM syndrome models.

Types	Examples	Characteristics	Application
*TCM etiology and pathogenesis *			
Four diagnostic methods	Red tongue model induced by long-term heat drugs treatment; [17] Thin white greasy tongue fur treated with alcohol, overeating, and eating disorders [18]	*Advantage*: Under the guidance of TCM theory; Simulating the TCM clinical etiology and pathogenesis; Symptoms are similar to human; Much closer to the TCM syndrome model *Disadvantage*: Single intervention factor; TCM syndromes are separated from diseases; Difficult to control the TCM pathogenic factors and TCM syndrome model it causes; Without specific and accurate pathological changes; Lacking stability and poor repeatability	The theory of etiology and pathogenesis of TCM study; TCM therapeutics study
Eight-principle	*Heat* syndrome induced with excessive exercise after oral administration of heat drugs [19]		
Qi-Blood-Liquid-Fluid pattern identification	*Qi*-deficiency model induced by starvation [20] Blood-deficiency syndrome treated with ^60^CO *γ*-ray radiation or cyclophosphamide [21]		
Viscera syndrome types	Heart-Blood-Stasis syndrome induced by coronary artery ligation or continuous injection with isoprenaline [22, 23]; Spleen-deficient syndrome induced with low-protein diet and lapactic herbs or subcutaneous injection of reserpine [24] Liver-*Qi* depression model established by bandaging or irritating animals [26–28] Kidney deficiency syndrome induced by excessive adrenocortical hormone [5]		

Based on Western medicine etiology and pathology	Stomach ache induced by formalin or salicylic acid (p.o.) [25]; Spleen asthenia syndrome treated with reserpine [24]; Blood-Stasis syndrome induced by ameroid constriction of a coronary artery [26];	*Advantage*: Specific and accurate pathological changes; Indicators are objective, standardized and can be quantified; Highly reproducible; Easy to establish; *Disadvantage*: Without clinical etiology evidence; Lacking relationship with TCM theory; Inappropriate tongue and pulse presentations	Pathology study; Mechanism of drug effect on disease model; Herb screening

Integrated with traditional Chinese and Western medicine	Heart-Blood-Stasis syndrome model basis on type 2 diabetic animals supplemented with prednisolone and adrenaline injection [27]; Congealing-*Cold* with Blood-Stasis syndrome model treated with adrenaline injection plus ice-water bath [28]	*Advantage*: Combine disease and TCM syndrome on the same animal model; Basis on stable and reliable disease model; Highly reproducible; Discussing the relationship between pathophysiology of disease and characteristics of TCM syndrome; Combining TCM theory and experimental method with macrocosm and microcosm unifies in an animal model; To diagnose a disease first then to identify the TCM syndromes *Disadvantage*: Application limitations; Affected by environment, species of animal and animal individual differences	Pharmacodynamics study; Mechanism of drug effect on disease model; A link between TCM theory and clinic; Essential of *Zheng* study

Genetically engineered animals	Phlegm-Stasis syndrome (*ApoE* gene knockout mouse) [29]; *Chan-*syndrome (Knockout mice of *Nurrl *gene); [30] *Yin*-deficiency and Blood-Stasis syndrome in diabetes (*db/db* mutant mouse); [31] Arthromyodynia (*Bi*-syndrome) (Abb knockout/transgenic HLA-DR4 mice) [32]	*Advantage*: Stabilization; High fidelity; Hereditary; High consistency with human disease *Disadvantage*: Limited categories (only mouse); Expensive; Difficult technology and complicated methods;	Pharmacodynamics study; Mechanism of drug effect on disease model; Drug screening; Pathogenesis study of hereditary disease, immunodeficiency disease, tumor, TCM syndrome animal model of insufficient natural endowment;
